# Cenobamate significantly improves seizure control in intellectually disabled patients with drug-resistant epilepsy and allows drug load reduction

**DOI:** 10.3389/fneur.2023.1209487

**Published:** 2023-07-17

**Authors:** Anna-Lena Friedo, Benedikt Greshake, Konstantin L. Makridis, Hans-Beatus Straub

**Affiliations:** ^1^Epilepsy Center Tabor, Bernau bei Berlin, Germany; ^2^Department of Pediatric Neurology, Charité – Universitätsmedizin Berlin, Berlin, Germany; ^3^Center for Chronically Sick Children, Charité – Universitätsmedizin Berlin, Berlin, Germany; ^4^German Epilepsy Center for Children and Adolescents, Charité – Universitätsmedizin Berlin, Berlin, Germany; ^5^Institute of Cell Biology and Neurobiology, Charité – Universitätsmedizin Berlin, Berlin, Germany

**Keywords:** cenobamate, drug resistant epilepsy, seizure freedom, intellectual disability, drug load, anti-seizure medication, outcome

## Abstract

**Introduction:**

Epilepsy patients with intellectual disability often suffer from drug-resistant epilepsy (DRE), which severely affects patients’ quality of life. Cenobamate (CNB) is a recently approved novel and effective ASM that can achieve high rates of seizure freedom in previously drug-resistant patients.

**Methods:**

We performed a retrospective data analysis of the first patients treated with CNB at a single center. Outcome and treatment response were assessed at two different time points, and ASM burden was calculated.

**Results:**

A 12 patients (7 males and 5 females) began treatment at a median age of 43 years, six of whom had developmental and epileptic encephalopathies. Prior to treatment with CNB, patients had tried a median of 13 different ASM. At the start of CNB therapy, patients were taking a median of 3 ASM. Treatment outcomes were available for 11 patients. After the first follow-up period (median 9 months), 55% of patients showed a significant seizure reduction of more than 50%, with three patients showing a reduction of more than 75% (27%). One patient achieved complete seizure freedom, while one patient did not respond to treatment. These response rates were consistently maintained at second follow-up after a median of 22 months. Ten patients (83%) reported adverse events (AE), the most common of which were dizziness and fatigue. No cases of drug reactions with eosinophilia and systemic symptoms (DRESS) were observed. The majority of AEs were mild and resolved over time. In addition, most patients were able to reduce their concomitant ASM.

**Discussion:**

Cenobamate has been shown to be an effective ASM in patients with DRE and in patients with intellectual disabilities. After more than 1 year of treatment with CNB, close monitoring and management of drug–drug interactions may reduce enzyme-inducing ASMs and lead to better long-term outcomes. With CNB treatment, many patients can achieve a reduced overall drug burden while maintaining a reduction in seizures.

## Introduction

Epilepsy is one of the most common neurological disorders. The primary objective of treatment with anti-seizure medication (ASM) is seizure freedom, with good tolerability of the prescribed ASM (1). However, in almost one third of patients seizure freedom is not achieved (2). Uncontrolled seizures of patients with drug resistant epilepsy (DRE) can result in cognitive deterioration. In individuals diagnosed with developmental and epileptic encephalopathies (DEE), the developmental delay or regression observed is related to the epileptic activity itself and the underlying etiology (3, 4). In Germany up to 1.5 million individuals have an intellectual disability, and 330,000 are projected to suffer from epilepsy (5). A systemic undertreatment of the intellectually disabled has been recognized, but targeted efforts have been made to remedy this in Germany over the past 5 years (6). Thus, there is a high need for improvement in the care of adult and pediatric epilepsy patients with intellectual disability especially as not all patients with focal epilepsy can undergo epilepsy surgery (7–12).

Neurologists face challenges in optimizing ASM management in patients with intellectual disability, resulting in suboptimal treatment outcomes (13). Factors such as infrequent patient visits, difficulty in assessing seizure burden and adverse drug events, and challenges in determining the severity of adverse drug events in patients with intellectual and language disabilities contribute to this problem (14–16). In addition, polypharmacy is common in these patients, complicating ASM adjustments. The treatment gap is exacerbated because tertiary care centers, where these patients are often treated, may have limited capacity to manage complex cases.

Cenobamate (CNB) is a novel ASM which was approved in the EU and the US in 2021, and achieved high rates of seizure freedom in previously drug-resistant patients (17). Further real world data further strengthens the evidence of the efficacy of CNB (18). In regards to Cenobamate clinical trials, one of the three pivotal trials (NCT01397968) did not exclude patients with intellectual disabilities or DEE (Dravet or LGS) while two others (NCT01866111 and NCT 02535091) did exclude patients with presence or previous history of Lennox–Gastaut syndrome (19–21).

Recent case reports on patients with intellectual disabilities encourage the use of CNB in these populations (22, 23). Further CNB often allows a reduction in drug load in patients with DRE. However, there is still a knowledge gap on how to adjust concomitant ASM when initiating therapy with CNB. Here we describe the challenges and lessons learned in treating a cohort of the first 12 highly refractory patients with CNB, six of whom are DEE patients.

## Materials and methods

We conducted a retrospective data analysis of the first treated CNB patients at the Epilepsy Clinic Tabor Bernau (Epilepsy-Center Berlin-Brandenburg) which were the most refractory. Patient data was extracted using a standardized data sheet. For each patient, outcome and treatment response were assessed at two different time points, along with a calculation of ASM load. ASM load was calculated as a sum of the ratio of prescribed drug dose (PDD)/daily drug dose (DDD) for each ASM included in the treatment regimen. DDD corresponded to the assumed average maintenance daily dose of a drug for its main indication.^1^ Descriptive statistics were performed using R (version 4.2.1) and the packages *ggpubr, dplyr, reshape2, crosstable.* This was a retrospective assessment of anonymized patients data and as such no ethics approval was required.

## Results

We report on 12 adult patients (7 males, 5 females) who started treatment at a median age of 43 years (IQR: 16). Table 1 summarizes patient characteristics, drug load, adverse events, and treatment outcome. These patients had severe drug resistance as evidenced by a median of 13 (IQR: 2.25) prior ASMs, with each patient having received at least 10 different ASMs. Despite Vagus nerve stimulation in 8 of the 12 patients, seizure control remained inadequate. Six patients had DEE (Dravet syndrome: *n* = 1, Lennox–Gastaut syndrome: *n* = 5).

**Table 1:** Patient characteristics, adverse events, drug load, and seizure reduction outcome.

ID	Sex	Age	Diagnosis	No. of ASM before CNB	Concomitant ASM at start of CNB treatment	Adverse events	Drug load before CNB (g/d)	Outcome	Current drug load (g/d)
1	M	64	FE	13	PER, CLB, ESL	Potential cognitive deficits	3.25	75%	1.625
2	F	46	LGS	17	PHT, TPM, CLB, DZP	Gait disorder	5.49	50%	2.66
3	F	34	LGS	21	VPA, OXC, LAC, PER, CLB	Vertigo, fatigue, gait disorder	4.23	75%	2.25
4	M	30	LGS	10	VPA, FBM, CLB	Gait disorder	2.775	No effect	2.75
5	M	60	LGS	14	CBD, CLB, LEV, PHT, PER	Fatigue	5.44	75%	3.76
6	M	21	DS	10	LEV, CLB		2.66	75%	1.75
7	M	59	LGS	13	CLB, BRV, CBD, RUF, LTG	Fatigue	4.85	50%	4.10
8	F	44	RE	14	LTG, GBP, ESL		6.07	50%	5.35
9	M	42	FE	11	OXC, TPM, PER	Visual blurriness, fatigue	6.36	50%	6.66
10	M	31	FE	12	CLB, PHT, CBD, PGB, RUF	Vertigo	5.41	50%	3.66
11	F	44	FE	13	ZNS, CBZ, CNZ	Fatigue	2.10	Loss to follow-up	Loss to follow-up
12	F	39	FE	14	LAC, BRV	Nightmares, vertigo	3.00	Seizure free	3.33

Diagnosis: DS, dravet syndrome; FE, focal epilepsy; LGS, lennox–gastaut syndrome; RE, rasmussen’s syndrome; BRV, brivaracetam; CBD, cannabidiol; CLB, clobazam; CNB, cenobamate; DZP, diazepam; ESL, eslicarbazepine acetate; f, female; FBM, felbamate; GPB, gabapentin; LEV, levetiracetam; LTG, lamotrigine; m, male; OXC, oxcarbazepine; PER, perampanel; PHT, phenytoin; TPM, topiramate; VPA, valproic acid.

**Figure 1:**
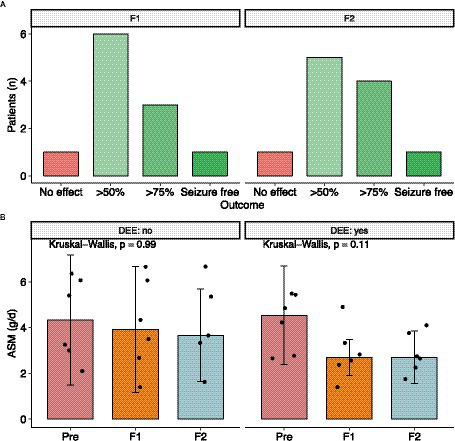
Outcome after CNB initiation. **(A)** Seizure outcome after first and second follow up. **(B)** Dose Reduction of concomitant ASM was possible in almost every patient. However mostly in patients with developmental and epileptic encephalopathy the total drug load was reduced. DEE, developmental and epileptic encephalopathy.

At the start of CNB therapy, patients were receiving a median of three ASM (IQR: 1.25), with clobazam being the most used (*n* = 8), followed by perampanel (*n* = 4). Titration was performed according to the CNB Summary of Product Characteristics. Patients received high doses of concomitant ASM, with a median of 4.54 g/d (IQR: 2.51, range = 6–1, *n* = 12). Follow-up was assessed at two different time points with a median follow-up of 9 months (IQR: 2.25) and 22 months (IQR: 2, range: 19–24, n = 11). Of the 11 patients with available treatment response data, six patients showed a significant seizure reduction of more than 50% (55%), three patients showed a reduction of more than 75% (27%), one patient was seizure free, and one patient did not respond to treatment (Figure 1A). As a result, 91% of patients with severe DRE responded to treatment at the first follow-up visit. These treatment response rates remained constant, except for one patient who showed a greater than 75% improvement in seizure reduction. All other patients maintained the same level of response (Figure 1B). There was no difference in treatment response between patients with a Vagus nerve stimulator (*p* = 0.5758; Fisher’s exact test for count data) and patients with a DEE (*p* = 0.6104; Fisher’s exact test for count data).

During CNB titration, AE were reported in 10 out of the 10 patients (83%). The most common AEs observed were dizziness and fatigue. Notably, none of the patients experienced drug reaction with eosinophilia and systemic symptoms (DRESS). Most of the AEs were mild in nature and resolved over time or due to concomitant ASM being reduced. Especially patients with high levels of N-desmethyl clobazam experienced adverse drug events such as unsteady gait and dizziness. At the last follow-up, seven out of eight patients had successfully discontinued clobazam. Overall drug load could be reduced from 4.3 ± 1.49 to 3.5 ± 1.69 and 3.44 ± 1.53 at last follow up (Kruskal Wallis test, *p* = 0.34) (Figure 1C). Looking at other ASM such as voltage-gated sodium channel (VGSC) inhibitors (ESL, OXC, LAC, LTG, and CBZ), four patients had these ASM eliminated, one patient had the dose increased, one patient had the dose halved, and one patient had the dose reduced by one-sixth. Of the four patients receiving PER, two patients had it eliminated, one patient had the dose increased, and one patient had the dose reduced by about half. Of the four patients taking an SV2A inhibitor, two had it eliminated and the other two had it reduced by about a third. For patients taking phenytoin (PHT), one patient had it eliminated, one patient had the dose reduced by half, and another patient had the dose reduced by about a third. In the case of patients taking topiramate (TPM), one patient had the ASM reduced by about a third, while the dose remained unchanged in the other patient. Valproic acid (VPA) was reduced in one patient and increased in the other. Two patients taking rufinamide had discontinued the drug completely, and one patient on felbamate had the medication reduced by one-third.

## Discussion

Here we report drug load, seizure activity and adverse drug events in 12 adult patients with highly drug resistant patients yielded promising results regarding clinical effectiveness. After more than 1 year, patients on CNB were able to reduce overall drug load with a corresponding reduction in seizures. Our real-world observations are essentially in line with the therapeutic effects and safety profile known from the RCT (19–21). When looking specifically at the DEE cohort, half of our patients showed a seizure reduction of more than 75%. We further report a patient with Dravet syndrome included in a series of four Dravet patients receiving CNB (24). It is unclear why CNB seems to be so effective in DRE. It may be due to its dual mechanism of action. It is thought to bind to voltage-gated sodium channels, blocking persistent sodium currents and reducing repetitive neuronal firing (25). CNB also binds to GABAA receptors and increases tonic inhibition (26). Whether it is this combination of CNB mechanisms of action or other novel mechanisms remains unclear and requires functional studies.

The treated patients reported improved quality of life due to a reduction in seizures. However, no formal assessment of health-related quality of life (HRQoL) was performed. It is important to note that in patients without sustained seizure control, adverse events are a particularly important determinant of HRQoL. Thus, further studies are needed to assess the effect of CNB therapy on quality of life. Because AE are important predictors, we have become increasingly aware of the importance of monitoring and managing drug–drug interactions when initiating CNB therapy. CNB itself is metabolized in the liver by glucuronidation (UGT 2B7; UGT2B4) and by oxidation via cytochromes P450 (CYP) 2E1, CYP2A6, CYP2B6. This however leads to several drug–drug interactions. CNB primarily inhibits CYP2C19 and is thus known to increase plasma concentrations of CYP2C19 substrates such as clobazam, phenytoin, and phenobarbital. These interactions can lead to the manifestation of AE early in titration, thereby necessitating prompt intervention to mitigate the risks associated with prolonged exposure. Of particular concern is the interaction between CLB and CNB, which has been shown to result in a marked increase in the plasma concentration of N-desmethylclobazam (N-CLB) by up to 500%. As we gained more experience in administering CNB, the interaction with clobazam emerged as a notable issue. Clobazam’s structure, confers preferential binding to the GABA _A_ alpha-2 receptor (27). N-CLB has a considerably longer half-life (59–74 h) than clobazam (36–42 h) and contributes to the drug’s spectrum of pharmacological effects. Administering CNB together with CLB results in an increase in plasma concentrations of both drugs, necessitating dosage reductions (28). Moreover, CNB raises the plasma concentration of N-CLB, which may increase the risk and/or severity of such as drowsiness, tiredness, drooling, constipation, and breathing difficulties (29). Therefore, to minimize the risk of AE, a proactive reduction in clobazam should be considered even at low doses of CNB, especially if somnolence and fatigue are observed. Also, cannabidiol (CBD) can both affect the plasma levels of clobazam and its metabolite, N-CLB. When combined with clobazam, CBD does not significantly affect the exposure of either drug but increases exposure to major metabolites of both compounds. In one case, a patient taking CBD, clobazam, and CNB experienced sharp increases in plasma levels of N-CLB. Ataxia or drunkenness including excessive sitting or lying down may be associated with intoxication at GABA receptors and may be a sign that clobazam needs to be reduced. For issues involving sedation, overexposure to co-medicated benzodiazepines is a reasonable assumption and reduction in benzodiazepines have been observed to lead to resolution of sedation, somnolence and fatigue in patients experiencing these effects (30). As mentioned CNB may also increases other CYP2C9 substrates. Phenytoin levels may increase up to two-fold thus it is recommended to adjust the dose during the titration period if blood levels of phenytoin are ≥15 pg./mL (31). Furthermore, phenobarbital should be proactively reduced at the start of treatment. For patients with intellectual disability in particular, listlessness or mutism in our experience is an indication of an acute need to reduce concomitant ASMs.

CNB is also a CYP3A4 inducer and thus may reduce ASMs that are metabolized by CYP3A4 such as carbamazepine, clonazepam or felbamate. Furthermore, lamotrigine and levetiracetam plasma concentration may also be reduced due to a yet unspecified mechanism, thus dose adjustment may also be relevant to increase concentration (31, 32). At higher doses of ASMs, pharmacodynamic effects result from ASMs with similar mechanisms of action. For example, the pharmacodynamic interaction between CNB and other sodium channel blockers may result in ataxia, diplopia, and dizziness. Due to pharmacodynamic effects as treatment with another sodium channel blockers results in the highest frequency of AE when initiating therapy with CNB (33). In such cases, a reactive reduction in concomitant ASM should be considered. In general, in patients with other sodium channel blockers, reduction of these may be necessary. However, in the long term with sustained seizure freedom/reduction, CNB may potentially be the only sodium channel blocker.

## Conclusion

Cenobamate has demonstrated efficacy as an ASM, as evidenced by real-world data in patients with highly DRE. Our findings highlight the importance of optimizing the timing of titration of non-effective ASMs, which can be achieved through careful consideration of caregiver reports and behavioral cues, particularly in patients who may be nonverbal. The use of adjunctive CNB in DRE patients has the potential to improve quality of life, making it a viable therapeutic option to achieve this goal.

## Data availability statement

The raw data supporting the conclusions of this article will be made available by the authors, without undue reservation.

## Ethics statement

Ethical review and approval was not required for the study on human participants in accordance with the local legislation and institutional requirements. Written informed consent for participation was not required for this study in accordance with the national legislation and the institutional requirements.

## Author contributions

A-LF, BG, and H-BS contributed to the conception, design of the study, and acquisition of the data. A-LF, BG, KM, and H-BS organized the database and analyzed the data. All authors discussed the results, revised the first draft, and contributed to the final manuscript.

## Funding

This research was undertaken, in part, thanks to scientific writing funding from Angelini Pharma GmbH, Munich, Germany. The funder was not involved in the study design, collection, analysis, interpretation of data, the writing of this article, or the decision to submit it for publication.

## Conflict of interest

A-LF has received funding for speaking engagements from Angelini Pharma. BG has received funding for speaking engagements from Angelini Pharma. KM served as a speaker and received travel expenses from Angelini Pharma and Jazz Pharmaceuticals.

The remaining author declares that the research was conducted in the absence of any commercial or financial relationships that could be construed as a potential conflict of interest.

## Publisher’s note

All claims expressed in this article are solely those of the authors and do not necessarily represent those of their affiliated organizations, or those of the publisher, the editors and the reviewers. Any product that may be evaluated in this article, or claim that may be made by its manufacturer, is not guaranteed or endorsed by the publisher.

## Footnotes

^1^ https://www.whocc.no/atc_ddd_index/
